# Genotyping Genebank Collections: Strategic Approaches and Considerations for Optimal Collection Management

**DOI:** 10.3390/plants14020252

**Published:** 2025-01-17

**Authors:** Noelle L. Anglin, Peter Wenzl, Vania Azevedo, Charlotte Lusty, David Ellis, Dongying Gao

**Affiliations:** 1 United States Department of Agriculture Agricultural Research Service Small Grains and Potato Germplasm Research, Aberdeen, ID 83210, USA; dongying.gao@usda.gov; 2 Centro Internacional de Agricultura Tropical (CIAT), Km 17 Recta Cali-Palmira, Palmira 763537, Colombia; p.wenzl@cgiar.org; 3 International Potato Center (CIP), Lima 15023, Peru; vania.azevedo@cgiar.org (V.A.); d.ellis@cgiar.org (D.E.); 4 CGIAR Genebank Initiative, The Alliance of Bioversity International and the International Center for Tropical Agriculture (CIAT), Via di San Domenico, 1, 00153 Rome, Italy; c.lusty@cgiar.org

**Keywords:** genotyping, genebanks, in vitro, clonals, and genetic identification

## Abstract

The maintenance of plant germplasm and its genetic diversity is critical to preserving and making it available for food security, so this invaluable diversity is not permanently lost due to population growth and development, climate change, or changing needs from the growers and/or the marketplace. There are numerous genebanks worldwide that serve to preserve valuable plant germplasm for humankind’s future and to serve as a resource for research, breeding, and training. The United States Department of Agriculture (USDA) National Plant Germplasm System (NPGS) and the Consultative Group for International Agricultural Research (CGIAR) both have a network of plant germplasm collections scattered across varying geographical locations preserving genetic resources for the future. Besides the USDA and CGIAR, there are germplasm collections established in many countries across the world that also aim to preserve crop and plant collections. Due to the advancement of technology, genotyping and sequencing whole genomes of plant germplasm collections is now feasible. Data from genotyping can help define genetic diversity within a collection, identify genetic gaps, reveal genetic redundancies and verify uniqueness, enable the comparison of collections of the same crop across genebanks (rationalization), and determine errors or mix-ups in genetic identity that may have occurred in a germplasm collection. Large-scale projects, such as genotyping germplasm collections, require strategic planning and the development of best practices. This article details strategies and best practices to consider when genotyping whole collections, considerations for the identity verification of germplasm and determining genetic replicates, quality management systems (QMS)/QC genotyping, and some use cases.

## 1. Importance and Challenges of Genebank Management

Ex situ collections (genebanks) serve to maintain genetic diversity for current and future use in crop improvement, research, and educational programs. The maintenance of genetic resources into perpetuity, along with making these materials available for researchers worldwide, helps ensure food security for humankind’s future. This is especially critical due to global climate change and the need to produce more food to feed a growing world population, expected to reach nine billion by 2050, on the same or less area of land. More than high productivity, the need for new varieties that are more resistant to biotic and abiotic stress is urgent, as is the nutritional component. The development of resilient crops through the breeding and rapid identification of key traits is greatly needed to meet these demands. The underlying genetic diversity in plant germplasm collections is the lifeblood of plant breeding, making conservation of the genetic diversity of major crops critical, allowing for the mining of these collections for useful traits [1]. The germplasm from genebank collections is shared around the world for research, training, and breeding to virtually all countries under international treaties such as the International Treaty on Plant Genetic Resources for Food and Agriculture (ITPGRFA).

In the Second Report on The State of the World’s Plant Genetic Resources for Food and Agriculture [2], it was estimated that there are over 1750 genebanks worldwide holding approximately 7.4 million accessions, yet the report estimates that only 25–30% of these accessions are genetically unique. However, very few genebank collections have been systematically compared through genotyping or other methods to evaluate uniqueness or redundancies among and between collections. Direct comparison of accessions from one genebank to another is challenging due to issues such as incomplete passport data, incorrect taxonomic identification, accessions being renamed due to cultural and other reasons, genetic identity issues/mistakes in the handling and processing of the germplasm, different taxonomic systems being adopted by different genebanks, passport data not being publicly available, insufficient staff to take on this effort, genetic drift, as well as a host of other issues. Further, data resources at genebanks vary greatly, with some being supported by publicly searchable information management systems and associated tools to aid and track routine genebank operations, some not even having digitized information, and all combinations in between these two extremes. The level of public accessibility and sophistication for the database management of collections is often a factor of infrastructure, staffing, and historic or current funding levels.

Most germplasm collections are maintained as seed since it is the easiest, most efficient, and most cost-effective method for the maintenance of large plant collections. However, some crop germplasm collections are maintained as clones because the crop either does not readily produce seed (many crop wild relatives, CWRs, triploids, etc.), it produces recalcitrant seed (e.g., coffee, avocado, cocoa), or it is desired to maintain a specific, unique combination of alleles in their current allelic state, which cannot be maintained in botanical seed (e.g., cassava, potato, sweetpotato, etc.), as each seed will produce a new genotype and a new combination of alleles.

Unlike seed collections, which can, for the most part, be maintained in a freezer, clonal germplasm collections are either maintained in the field, greenhouse, in vitro, or, in some cases, cryopreservation. Each of these forms of maintenance comes with a unique set of challenges. Field and greenhouse collections are more vulnerable to loss due to natural attrition or insults from the environment (e.g., diseases, natural disasters, climate change, etc.). In vitro collections, on the other hand, are technically more difficult to maintain, requiring frequent maintenance and manipulation, which can increase the frequency of epigenetic or somaclonal variation, as well as human errors due to constant and reoccurring handling. Further, in vitro collections also have continual risks of contamination events (bacteria, mites, fungi, viruses, etc.) which can lead to the loss of valuable germplasm. Moreover, in vitro conservation requires the development of specific protocols for the different species and is sometimes genotype-specific. On the other hand, the advantage of in vitro collections is the ability to maintain accessions in their current allelic and disease-free state, greatly aiding in the ability to move germplasm around the world (https://cropgenebank.sgrp.cgiar.org/index.php/procedures-mainmenu-243/conservation-mainmenu-198/in-vitro-bank-mainmenu-200, accessed on 1 January 2025).

Although errors can occur in any germplasm collection no matter how it is maintained, as the material requires constant regeneration or handling to respond to germplasm distribution requests, in vitro collections typically require more consistent handling and manipulations by humans, which makes them more susceptible to errors. This situation occurs because cultures need propagating (subculturing of explants) every six to eight weeks when in active growth due to the depletion of nutrients and lack of space in the growing container [3]. This practice can over time introduce more errors than field collections of crops with long life cycles (e.g., trees), seed, and cryo collections; however, errors can occur in any germplasm collection no matter how it is maintained, as all material requires regeneration or handling to respond to germplasm distribution requests. Slow growth methods can be applied for in vitro cultures to reduce the metabolic activity or growth rate such as using additives for the standard media formulations like sorbitol or mannitol as osmotic regulators and using silver nitrate (AgNO_3_), which can improve regeneration and slow down growth. However, these chemicals can have effects on plant growth and development. Cooler growing temperatures, reduced lighting, and photoperiod manipulations can also be utilized; however, these strategies can only buy time before the culture will require propagating and need to be renewed. Some genebanks are investing in cryopreservation in lieu of slow growth methods to preserve materials for longer periods of time, theoretically centuries, to reduce the risk of changes, such as epigenetic changes, somaclonal mutations, and associated risks with the constant handling and manipulation of clonal crop collections (https://cropgenebank.sgrp.cgiar.org/index.php/procedures-mainmenu-243/conservation-mainmenu-198/in-vitro-bank-mainmenu-200, accessed on 1 January 2025).

Mistakes happen in all collections, and accessions can become mixed up, leading to genetic identity issues, which are a growing concern to curators and genebank managers, as the frequency of these mistakes is becoming more apparent with the advent and increased use of high-throughput genotyping. Of further concern is that these types of errors accumulate over time and thus, the older the collection, the higher probability of a greater error rates/genetic identity problems across the collection. Such historical errors are recognized as a management challenge since small events caused by changes in funding, genebank oversight and management, or the evolution of processes and data management can have exponential repercussions over time. The adoption of quality management approaches and much tighter processes can radically reduce the chance of mistakes being made, but they cannot undo those that occurred in the past. Errors in maintaining genetic identity have been previously reported as a problem in genetic stock collections [4]. Genetic identity has also been previously reported in some clonal collections. The International Potato Center (CIP) reported an error rate in genetic identity of 4.4% in 250 accessions (a small subset of the entire collection) from the cultivated potato collection where Single Nucleotide Polymorphism (SNP) genotyping demonstrated mismatches between a paired in vitro sample and the mother plant maintained in the field [5]. An expanded look at the genetic integrity of the cultivated potato collection (3860 accessions) at CIP uncovered an error rate of 19.9% [6]. The entire cultivated sweetpotato collection at CIP was evaluated for genetic identity using 20 Simple Sequence Repeat (SSR) markers along with evaluating morphological characterization in the field of paired samples, which found a 19.4% error rate in genetic fidelity among paired samples (in vitro to original mother plants) [7]. Another study evaluated errors in the sweetpotato breeding program at CIP and found a 27.7% error rate, which was suggested to have occurred when moving germplasm from in vitro, to a screenhouse and then the field [8]. Genotyping was employed in all of these studies, resulting in the elucidation of significant errors in germplasm collections, suggesting that these identity errors occur in virtually every genebank but are rarely assessed collection-wide due to the challenging logistics and cost of making these assessments. Even using low-density approaches or a small number of markers (SNPs, SSRs, Amplified Fragment Length Polymorphism [AFLPs], etc.) to evaluate collections for identity errors is sufficient to reveal problems, especially when comparing a field collection to in vitro as-paired samples.

Another way to evaluate genetic identity is to assess morphological characteristics and compare the current descriptor data to historical and/or available passport data or compare these to herbarium samples of the same accession, if available. Girma et al., [9] evaluated 3156 accessions of paired yam accessions and found a 20.6% error rate (not true to type), employing 53 morphological descriptors to assess genetic uniformity among paired samples. These errors in the yam collection were thought to have occurred via mislabeling, misreading labels, and material mix-ups during planting or storage. Automation such as barcoding implemented in handling and manipulation steps, the machine printing of labels, and a good quality management system (QMS) for tracking various workflows can greatly help reduce human-induced errors, but nothing is completely “error-free”.

Due to the high cost of systematically addressing identity issues across an entire collection, especially in large collections, few genebanks have had the funding or expertise to even start evaluating the genetic fidelity of their germplasm collections. Often, the funding for genebanks only covers the basic routine maintenance of the material, making additional projects not feasible. Genotyping and determining errors in seed collections with outcrossing species would be a significant challenge, as there is not a clear-cut, easy way to determine identity since each generation would produce new combinations of alleles. Another limiting factor is that not all genebank collections have paired samples, good historical data, or herbarium collections to use as a baseline to be able to determine if errors have occurred over time. However, it is important to recognize that genotyping a clonal germplasm collection or a seed collection that is a selfing species does set a baseline for the future and thus can play an important role in the maintenance of genetic integrity without having paired samples of accessions. Over time, the germplasm can be monitored to ensure that the genetic integrity of the accessions has not changed. If DNA fingerprints do not match after a few years or longer of routine maintenance, then errors in handling and manipulation have likely occurred. Another strategy that can be employed is to genotype every new germplasm acquisition at the point of entry into the genebank to establish a genetic baseline fingerprint for that accession; then, its genetic fidelity can be monitored over time as part of a QMS program. These data can be shared or made available as a molecular passport to other researchers, breeders, or other users requesting the material to help users monitor genetic identity in their programs.

Another concern in clonal genebanks, especially in vitro collections, is the production of somaclonal variants due to the artificial nature of tissue culture causing stress over time, which can produce changes in the DNA of a particular accession/genotype [10]. Epigenetics involving the adaptation of cells to a different environment can trigger switches in development, which may have undesired effects in the original objectives for the maintenance of tissue culture with a single genotype and result in a variety of phenotypic outcomes under the same culture conditions [11]. Both somaclonal mutations and epigenetics, or a combination of both, play a role, but mutations are not reversible, whereas epigenetic changes may or may not be reversible and generally are not transmitted through sexual reproduction [12]. A few studies have evaluated the frequency of somaclonal variants, but further studies are needed to adequately assess how frequently this occurs in genebank collections, and it is difficult to definitively identify the cause of a morphological change which has occurred in vitro. One genomics-based study of various potato species demonstrated an increase of transposons in the in vitro-maintained material [13]. However, little is known at this time of the overall effect or the meaning of this increase in transposons. Genotypes may play a role with the induction of these mutations with a higher frequency in the occurrence in some genotypes and/or species than in others. Further, propagation methods, growth regulators, culture media, the number and duration of subcultures, and starting tissue can also have an effect on induced mutations [12] or the frequency and rate of epigenetic changes, but further research is needed to understand their effect. Somaclonal variation, however, can be useful in research to generate novel variants or new varieties in plant breeding [12], but for the purpose of a genebank and maintaining the genetic fidelity of clones, it is an undesirable outcome.

The number of resources needed for the maintenance of clonal collections is high, especially when directly compared to the cost of routine operations of seed genebanks. The Consultative Group on International Agricultural Research (CGIAR) Genebank platform, in association with the Global Crop Diversity Trust (Crop Trust), has previously evaluated the routine operational costs of all 11 genebanks within the CGIAR (Internal paper—“summary of the findings of CGIAR genebank financial reviews and reported expenditures”, personal communications with Crop Trust). In 2020, the clonal crops represented only 5% of the 760,000 accessions maintained in the 11 genebanks yet required 36% of the total operational cost of the genebanks within the CGIAR. The clonal crops, trees, and tropical forage germplasm collections cost more than all of the seed genebanks combined, yet together, these collections only represent 12% of the total germplasm held within the CGIAR. Based on this CGIAR genebank cost analysis conducted before the COVID-19 pandemic and recent global inflation, which has led to increased costs, the average cost for maintaining a seed accession was USD 12.5 (not including the tropical forage collection in Ethiopia, which appears as an outlier), whereas the average cost for the maintenance of a clonal accession is tenfold higher at USD 127 for the CGIAR clonal collections based on the maintenance cost for collections at CIP (International Potato Center), IITA (International Institute of Tropical Agriculture), Alliance of Bioversity International, and CIAT (International Center of Tropical Agriculture), but not including Bioversity International, which is an outlier. The current cost analysis conducted at the International Potato Center (CIP) genebank showed that the cost is about USD 120/accession in Peru for the maintenance of a clonal accession. The clonal crops maintained in vitro within the CGIAR include Andean roots and tubers at CIP, banana/plantains at Bioversity International and the International Institute of Tropical Agriculture [IITA], cassava at Centro Internacional de Agricultural Tropical (CIAT) and IITA, potato and sweetpotato at CIP, and yam at IITA. Further, seed collections also require considerably less staff, with only one staff member needed/10,000 accessions for all collections over 100,000 accessions, no matter how large or complex the collection (Internal paper – “summary of the findings of CGIAR genebank financial reviews and reported expenditures”, personal communications with Crop Trust). Clonal crops, on the other hand, which are maintained as in vitro collections, require significantly more staff to keep these collections alive, with a median of three staff members required per 1000 clonal accessions (Internal paper—“summary of the findings of CGIAR genebank financial reviews and reported expenditures”, personal communications with Crop Trust). This is because compared to seed accessions, which can be regenerated on a 10-year cycle or longer and placed in a cold room for storage, in vitro cultures require constant monitoring for growth and potential contamination, routine subculturing with multiple tubes produced for conservation and safety backups, the production of appropriate media substrate, and the cleaning of culture tubes. Generally, a genebank can maintain ten seed accessions for every one clonal accession. A clonal collection is entirely regenerated every six months to two and half years, which is the maximum time that a clonal culture survives in vitro before requiring regeneration or subculturing. Therefore, the overall investment, staffing, and technicality required by clonal crops is quite different to seed crops and, thus, more labor-intensive and costly to maintain (Internal paper – “summary of the findings of CGIAR genebank financial reviews and reported expenditures”, personal communications with Crop Trust). Much of the costing of the CGIAR genebanks is based on defining a minimum standard of operation, which is guided by the FAO genebank standards [14]. However, international standards for clonal crops are not as developed as for seed operations between genebanks and still vary quite significantly because of different approaches for phytosanitary controls, the conservation of crop wild relatives, the use of field collections, and other aspects of genebank operation that relate to historical management, specific crops, and the environment. These costing data carried out by the Crop Trust, while now several years old, give an indication of the difference in scale of costs between clonals and seed collections.

## 2. Best Practices: Considerations Before Genotyping Any Samples

Genotyping whole germplasm collections is not a trivial task. Before getting started, there are several things that should be considered and decided prior to moving forward. First, an appropriate marker system needs to be chosen to evaluate the target genetic resource collection. This requires some guesswork, as technologies will change and become more efficient. For example, in the 1990s and early 2000s, producing sequence data from a single gene took quite a lot of effort and time, whereas now, whole genome sequence data are being rapidly produced at an affordable cost for many research groups. Currently, researchers most commonly reach for SNP markers (SNP arrays) or reduced representation sequencing methods (e.g., genotyping-by-sequencing GBS, restriction-site associated DNA sequencing RAD-seq, diversity arrays technology sequencing DArTseq, etc.), which call variants out of a subset of sequenced short reads from the target genome(s). However, before the affordability of high-throughput sequence-based systems, markers such as SSRs, AFLPs, restriction fragment length polymorphism (RFLPs), or random amplified polymorphic DNA (RAPDs), which were more labor-intensive to employ, were widely chosen to assess genetic variation among individuals [15]. It is likely that new marker systems will continually be developed, which will replace the ones commonly used today and be available for researchers for the more efficient, cost-effective capture of more data. Also, the number of markers available and capable of being interrogated in one pass will likely continue to rise. SNP markers are commonly used for genetic analysis, as thousands of SNPs can be evaluated in a single run. One potential drawback is that SNP arrays can lead to ascertainment bias, especially when the markers on an array were discovered from a small number of samples or samples that do not represent the broader population [16] and thus are missing the genetic diversity of the species. Arrays offer a quick, easy, affordable, and reproducible method for screening for known variants. However, they lack the ability to detect a significant proportion of rare variants and can be biased towards variants in the populations used to develop the respective array [17]. One significant challenge is that it can be significantly cumbersome to compare genotypes using different marker systems, which usually results in researchers having to start over in their genotyping efforts and select a method compatible with a particular marker that is the most efficient, inexpensive, reproducible, and familiar. That said, it is not impossible to compare across different marker systems, but generally, to do so requires intensive bioinformatics or a significant loss of data by finding commonalities between the different marker sets such that researchers generally wind up having to start over from the beginning. Further, once marker systems become obsolete, which happens over time, it is difficult to find service providers or consumables to keep generating data with that marker system, which makes future comparisons with genotyping data challenging. This has a significant impact on research and collection management because when a collection has been previously genotyped with one method, and this method becomes obsolete or no longer supported, and when new accessions are added to the collection, one has no other option than to move to a new marker system to ensure compatibility with new acquisitions. At this point in time, sequence-based markers should have greater longevity than other types of marker systems currently available. The recommendation is to utilize the latest and most reproducible technology as much as possible. Additionally, a sequence-based (future proof) technology that has been broadly adapted by multiple genebanks and collections is also recommended to help enable cross-genebank or cross-collection direct comparisons.

Another important consideration is the use of a laboratory information management system (LIMS) to manage the associated metadata produced in genotyping germplasm collections. Genotyping entire collections, especially germplasm collections with tens of thousands of samples, takes a considerable number of years to complete, even with a large labor force. Although it may have been possible at one time to manage this through excel or laboratory notebooks, this is no longer possible with the huge amount of data that are generated and the need to track and analyze information efficiently when multiple staff members are contributing and needing to access the data. Building a LIMS or purchasing an appropriate LIMS can greatly help in organization throughout the project and provide access to critical information for all of those working and investing in the genotyping effort. Managing data such as specific sample information (source tissue), sample collection dates, DNA extraction methods, staff that handled the extractions, dates of sample extractions, quantification and quality measurements of the extracted DNA, repeated or replicate samples with their associated information, and associated genotypic information performed in house in leu of a service provider can quickly become an enormously large dataset which is unwieldy to manage without an adequate LIMS. Accurate sample tracking is a critical aspect, as clonal and seed genebanks often have multiple sources of samples (in vitro, cryo, field collections, greenhouse, herbariums, seed inventories from different regeneration cycles, etc.), and it is critical to track the source sample of every DNA extract to address questions which arise later, especially questions related to genetic identity. As stated earlier, it is possible that a plant accession can be stored in vitro, as well as in the field, with both samples having the same accession number, but when genotyped, they may or may not be the same genotype due to mixing/handling errors. Therefore, tracking and recording sample sources is a must. Girma et al., [18] produced a guide which details sample collection, tracking, and DNA extraction in cassava, with good recommendations on managing the process with step-by-step instructions. Investing in barcoding for all aspects of sample tracking that interact directly with the LIMS is highly desirable and helps reduce human-induced errors such as reading handwritten labels. Handheld barcode readers and pocket printers can help staff move quickly through the processing of samples from the point of collection to DNA extraction, all of which should directly interact with the LIMS in real time to track all manipulations, dates, times, and staff members performing the lab task(s). These systems also have error checks where alarms trigger an alert to the user when scanning multiple inventories of the same accession and the barcode reader finds that they do not match. Having easy access to information for everyone involved in the project helps sort out potential problems that may arise and also allows for the tracking of the overall progress of the project.

DNA extraction methods, if not already known, should be tested and evaluated to ensure that high-quality and sufficient quantities of DNA can be obtained for the marker system chosen to genotype the collection. Service providers and marker types all have different requirements for DNA quantity and DNA quality. Having a surplus of DNA or conducting multiple extractions in case there are issues in the shipping of the DNA to the genotyping service provider should also be considered, as packages go missing, seals on plates/tubes may at times have issues in the shipping process, and extreme fluctuations in temperature can affect the extracts. Also, some DNA extraction methods do not work equally well on all plant species. For example, plants high in phenolics or polysaccharides can make DNA extraction more challenging. Polysaccharides often co-precipitate with genomic DNA, giving an extract a viscous consistency. Further, terpenoids and tannins oxidize rapidly after the release from leaf tissue and irreversibly bind to the phosphate backbone of DNA [19]. Frequently, labs use either hexadecyltrimethylammonium bromide (CTAB)-based methods [20], modified CTAB methods which include polyvinylpyrrolidone (PVP) [19,21], or commercially available kits. Commercial kits are advantageous as they are quality-controlled and simplify many of the extraction steps by using binding and purifying columns instead of chemical separations; however, they can be cost-prohi-bitive compared to CTAB-based methods especially for big collections with thousands of accessions to extract. Further, CTAB methods can at times result in superior DNA quality when DNA is extracted across a wide taxonomic range of CWRs. The different extraction methods used need to be tested on the species of interest to ensure that consistent DNA can be obtained with A260/280 ratios at approximately 1.8–2.0 and that the DNA is not extensively sheared in the process. It is often of value to run samples with a particular DNA extraction method on a small scale with the marker system of choice before moving into genotyping the entire collection to ensure its efficacy and a good return on the investment. As mentioned before, barcoding and LIMS can be critical tools to help manage and label the extracts generated to genotype a germplasm collection.

LIMS can also be an important factor to consider in helping with disruptions that occur due to transitions of staff. Genotyping projects for entire germplasm collections can take years to complete and, in that time, it is inevitable that changes in staffing will occur. Time will be required to get new staff trained in the workflows, LIMS, data collection, DNA extraction, genotyping analysis, and so on. A LIMS can help make these transitions more seamless by providing a system where all associated information can be accessed and reviewed in one place and in real time, rather than relying on lab notebooks and deciphering handwriting or multiple excel files. New staff can review the LIMS to see what data are collected and the quality of the collected data to help understand their role in the genotyping project. Turnovers in staffing can significantly slow down the progress of sample collection, DNA extraction, and genotyping since time is required to advertise, rehire, and train new staff. It is wise to keep in mind that these issues can and will occur, ultimately delaying project deliverables. A related consideration is identifying collaborators or current staff who are adept at analyzing all of the collected genotyping data. Bioinformatics support is of paramount importance when managing large datasets, along with having the basic knowledge of how to analyze and interpret the data. For example, experience working with software programs that can handle large datasets of thousands of individual samples and genetic markers is critical because Excel and other standard programs may be unable to handle this amount of data.

Replications of the genotyping data is another important consideration. Technical and biological replicates should be built into all genotyping projects. Technical replicates are samples that are genotyped more than once—e.g., a particular DNA extract is repeated in the genotyping process. Technical replicates should be submitted both within and between genotyping runs to assess differences among different runs of a particular marker system. A biological replicate is a sample that is recollected and whose DNA is re-extracted, and this second DNA is re-genotyped—basically starting all over from the beginning. Replications allow for understanding errors that may be occurring within a genotyping project. They also help define the error threshold of each technology, allowing for the differentiation between genetic differences among samples and artifacts of the technique. This strategy helps flush out human errors as well as machine-induced errors, both of which can and do occur. Technical or biological replicates that produce radically different genotyping results suggest that errors are occurring either at sample collection, DNA extraction, or preparation of the DNA for genotyping (arraying samples into a plate for genotyping or other manipulations). Good quality control measures are needed to reduce human errors.

All technologies have some inherent error rate (typically ~1–3%), and by including technical and biological replicates, one can reveal the extent of these errors and the approximate rate of error and account for this in the data analysis. Replications are of particular importance if one wants to evaluate genetic duplicates within the germplasm collection, as it is challenging to know where to set a threshold for calling and purging duplicates based on genetic similarity. A recent study in cassava used a series of different replications (technical and biological replications including collecting individual accessions obtained from different conservation units, DNA extracted from the same individual, repeated genotyping of the same DNA sample) to reduce miscalling errors, DNA extraction errors, and traceability errors to facilitate the identification of genetic duplicates [22]. These built-in replications provide a baseline for the error rate of the machine/technologies being used. Therefore, if, for example, the technical replicates of the same sample show an average of a <3% difference in total genotyping calls across the same sample genotyped multiple times, then a cutoff threshold can be set at 3% for determining genetic duplicates. Any samples that have a 3% or smaller genotypic difference can be considered genetic duplicates and may be considered for archival or possible elimination. Anything greater than a 3% difference can be considered unique. However, it is important to highlight that, for polyploid species, some markers commonly used do not provide allele frequency but the presence and absence of data instead. Thus, sometimes samples that are within the threshold cutoff are genetically different, but the allele dosage was not obtained, leading to a lower estimation of diversity. Because of this issue, morphological characterization becomes highly relevant and needed.

Confirming and identifying genetic duplicates can be challenging due to differences in the marker density being employed versus the size of the genome, epigenetic effects, and subtle morphological differences between putative duplicates, which all need to be considered. All of these factors can occur between two accessions with highly similar genetic profiles yet have some differences in their morphology. For example, sweetpotato accessions genotyped by high-density DArTseq and 20 SSR markers demonstrated matching genetic fingerprints suggesting genetic duplication; however, in some cases, field evaluation showed morphological differences, making calling these accessions “true duplicates” impractical (unpublished data). Therefore, it is suggested, at least with clonal crops, to assess the morphology side-by-side with the putative genetic duplicates to capture subtle variation that is not revealed in genotyping alone prior to making any decision on the fate of potential duplicate accessions. These decisions may need to take into consideration the current resources and whether it is worth removing or archiving very similar material to make space for new acquisitions that are more divergent.

Biological replicates, on the other hand, not only help with setting the threshold of machine-level errors but also help determine if mistakes are occurring during sample collection and/or DNA extraction if the genotypes of the biological replicates do not match. It is important to keep in mind that errors can and will occur at any point in the workflow, and basic quality control (QC) such as barcoding can help reduce these errors overall but likely will not eliminate all errors completely.

Understanding “the good, the bad, and the ugly” of genotyping data is also a key concern which needs to be considered. Researchers need to be able to quickly know if a sample and its associated genotyping data are good or need to be repeated. When DNA quality is poor (low-yield, fragmented, etc.), typically the genotyping data will be affected. Samples producing a low number of markers, unscorable portions, or an unusually low number of sequence reads are examples of factors likely to result from poor DNA quality, low DNA quantity, or other technological issues surrounding the platform being applied. Some markers may not work well in certain genotypes and may need to be eliminated. There may be a large production of null alleles produced, which also needs to be considered. Markers can be monomorphic across most or all of the samples, making the marker unsuitable for discrimination purposes and most other genetic applications. Setting thresholds for samples and marker (meta) data helps remove subjectivity in decision-making for determining where the data are good or bad. For example, a common threshold and filtering criterion for marker data is 10% or less missing data and minor allele frequency (MAF) > 0.05 [6]. This strategy works well for breeding lines, where the genetic diversity is not expected to be so high, but for genebank materials with a high range of diversity, there is always a risk of eliminating real diversity among samples. Generally, the same is true for samples. If a given sample contains a high proportion of missing data, it likely needs to be checked for DNA quality/quantity and repeated. However, frequently in reduced representation sequencing methods, genetically distinct/distant accessions will tend to produce a higher percentage of missing data due to the reduced overlap of genome representations, which needs to be considered when applying thresholds for sample filtering. This makes DNA quality especially important to genebank analysis so these small differences can be better captured and not expected to be caused by poor DNA quality.

Another consideration in large genotyping projects is depositing the data in an open-access database and the future curation of the data. Many donors and journals require that all data be made available in open access. Quite a few platforms already exist for making data available, so there is no need to develop a new system for a particular project. Further, making data readily available helps align a research study with the FAIR (findability, accessibility, interoperability, and reusability) Data Principles [23]. A straightforward way to comply with the open access of genotyping data is to submit the data to the journal as “supplemental material”, which allows all researchers to gain access to the data, assuming the article is published in open access. Other options include crop-specific databases that generally house genomics data, linkage maps, and genotyping data on a single platform. Many institutions have also embraced databases for the open access of data. For example, the CGIAR uses dataverse (https://dataverse.org/, accessed on 1 January 2025), which is an open-source web application. Other sites are available such as figshare (https://figshare.com/, accessed on 1 January 2025) and Mendeley Data (https://data.mendeley.com/, accessed on 1 January 2025). Digital object identifiers (DOIs) can also be assigned to datasets, as well as different licensing schema such as a creative commons copyright license, which allows an author to give the right to share, use, and build on their work noncommercially (https://creativecommons.org/licenses/, accessed on 1 January 2025).

### 2.1. Genotyping Collections for Quality Control

Because genetic identity problems can quickly accumulate over years from processing and handling material in a collection, genebanks use multiple ways to monitor genetic integrity. Clonal collections, collections of seeds from selfing crops, or particularly expensive-to-conserve crops may benefit from investing in a Quality Management Systems (QMS) genotyping. If a genebank has an average of 1% labelling or genetic identity error rate/year, which may be a conservative error rate, over time, this could equate to a 10% error rate in a collection in 10 years for an in vitro collection that must be replicated every year. Of course, this is just an approximation and depends on the average error rate and how often the collection is manipulated and turned over/regenerated. Seed collections that are not regenerated so often should, in theory, have significantly lower error rates over time, but of course, all errors should be avoided to maintain the integrity of the collection.

A large identity verification project was carried out at CIP after evidence was found of genetic identity errors in the collections. All field and in vitro paired samples of the same accession were genotyped and phenotyped to confirm their identity. Herbarium specimens, cryopreserved accessions, historical data, and voucher samples can also be used as a reference, if available, to check for genetic integrity or provide a baseline for genetic integrity. If samples did not have a field and in vitro counterpart to compare directly, which was the case in about 45% of the sweetpotato collection at CIP, then genotyping and phenotyping were carried out on the single sample of the accession to generate baseline data for the accession for any future assessments or questions of genetic identity. In these cases of unpaired accessions, the accession was grown out in the field to measure their characteristics, and comparisons were made with any passport and evaluation records to determine if the sample identity appeared to be correct and to act as a baseline from which to monitor the genotypic and phenotypic data in the future. Once the entire cultivated collection was genotyped and phenotyped, any not-true-to-type accessions were replaced and corrected. These data provide a baseline for the ongoing quality control of the collections into the future. Such a quality control strategy works well for clonal collections and relatively well for seed collections that self-pollinate, since segregation can result in slight changes in allele frequencies over time on heterozygous loci. A separate strategy would be needed for seed collections that outcross due to generating new variability in each regeneration.

The basic premise of the QC strategy was to use the collected genotypic data to randomly monitor the collection for errors. This entailed randomly sampling 10% of the subcultured potato and sweetpotato in vitro accessions each year for genotyping and verification against previously collected genotypes. (DArTseq was used for sweetpotato and Illumina Infinium SolCAP potato SNP Array was used for the potato samples). The majority of the randomly chosen accessions matched the original fingerprint collected. However, ~1% of the samples randomly chosen in both crops had mismatched fingerprints. This was a surprising result since all systems and manipulations within the genebank incorporated barcoding and digitally printed labels and there was thus an assumption that human induced errors would be reduced or non-existent. Tracking and tracing occurred at each and every step throughout all workflows with data collected digitally; however, many steps in the in vitro process are manual. This exercise proved and cemented the notion that a 0% error rate may be unrealistic as long as processes cannot be completely automated.

Every time a sample is handled and manipulated, there is the potential for an error to occur despite a well-developed and audited QMS system for all in vitro processes. Some of the reasons identified for the potential generation of errors are a high turnover rate of staff and repeated tasks—doing daily propagation and manipulation of the plant material (in vitro or in the field). Moreover, other issues arise such as the tendency for humans to go on autopilot or having others interrupt their train of thought during the handling of the accessions. Further, due to the volume of material needing routine propagation in larger genebank collections, four to eight technicians propagating all day long, five days a week, can likely also lead to the generation of mistakes. Distractions can be a constant source of errors in any process, which is hard to control from a managerial perspective because this is internal to each staff member.

Quality control based on genotyping a random selection of regenerated samples can quickly indicate the level of error occurring in processes and further determine if corrections are needed. CIP was in a unique position to monitor genetic integrity from their previously collected genotyping data [5,6,7]. If errors could be identified and corrected on an annual basis through QMS genotyping, then some of the mixed-up accessions would be corrected and not compounded over the years and ideally not be distributed to the requestors incorrectly. Further, if there are questions on identity arising from requestors or observations of the accession not appearing as expected, genotyping of a few samples could be undertaken to quickly sort out the questions on its identity. The big disadvantage to the overall strategy is that if errors are always occurring, then random genotyping is a constant need, which results in a never-ending process of error sorting, which has cost implications associated with it, and very few donors are interested in supporting this activity. For example, the cost of introducing a sample from the field to replace an in vitro accession to correct an identity error was ~USD 450 for the introduction and then phytosanitary cleaning of that sample, and an added cost of ~USD 550/sample was also required, ultimately creating a bottleneck in the phytocleaning pipeline. (Costs listed are operational costs at CIP and may be different for genebanks in other locations due to staff salaries, supply costs, etc.). The costs to “fix” an identity error by replacing a field or screenhouse accession with an in vitro accession are not as prohibitive (USD 20/sample) since no phytosanitary cleaning is required.

Maintaining multiple sources of material in different conservation forms (in vitro, field, greenhouse, cryo, herbarium, etc.) as a genetic reference to correct identity errors can significantly add to the overall operation costs of a genebank but can be useful in sorting out identity issues. On the other hand, the acquisition of new genetic material can be time-consuming (obtaining country permission and the necessary permits) and costly; thus, having these backups might be necessary, since going back to the original donor or collection site may not be a possibility. In some cases, these materials do not even exist anymore in their original site. While it is important to identify errors rapidly, clean them up before they compound, and not have potential mistakes being distributed out to users, causing embarrassment for the genebank, it also comes with additional operational costs (having multiple samples in different forms along with repetitive annual genotyping). Furthermore, the idea of getting to a zero percent error rate is unrealistic, even with so much automation in place. The automation helps to reduce the errors but not completely eliminate them. CIP has had barcoding in every workflow for over 15 years, but errors still occurred based on QMS genotyping results.

### 2.2. Genotyping for Rationalization Between Collections

Another strategy for genebanks to take in order to possibly alleviate some of these pitfalls is to rationalize between collections of the same crop via genotyping and comparing passport data among the collections. While CIP was genotyping and analyzing data from the entire in-trust global potato and sweetpotato collection (held in trust for humanity under the International Treaty on Plant Genetic Resources for Food and Agriculture {ITPGRFA]), they collaborated with scientists at the USDA on the sweetpotato collection [7], Seed Savers on their potato collection in the USA [6], and the Center for Pacific Crops and Trees (CePaCT) on their sweetpotato collection [unpublished data]. Once the genotyping data were collected from the collections, comparisons were made on areas of duplication and phylogenies were produced to evaluate diversity and potential genetic gaps. Identity could also be evaluated by looking at accessions that were in common between the two accessions. There was some overlap between the USDA, CePaCT, and CIP sweetpotato collection, as material had been shared between these genebanks over time. Interestingly, no overlap was found between the Seed Savers collection and CIP, with these two collections being quite unique [6].

CIAT (Colombia) has been working on a similar strategy with IITA (Nigeria) to genotype the two cassava collections in order to look at redundancies and genetic differences among the two collections. Finding genetic redundancies between the two cassava collections could help to correct future genetic identity errors, as a sample in question at one center could be obtained from the other to ensure it is still correct when a question of identity arises. Moving germplasm from one genebank to another is less prohibitive or difficult than recollecting material from its country of origin. Safety backups of clonal collections is another potential way to accomplish the same outcome by assessing if the safety backup sample has the same genetic fingerprint as the sample in question. However, a problem with clonal collections is that if backed up in vitro, which is often the case, they require the constant propagation and replacement of in vitro safety backups (approximately every year to two). Hence, if an error occurs early in the safety backup cycle, then the error is also sent to a safety backup site (national or international), and the backup will contain the same error as the main collection. Obviously, safety backups in cryopreservation would alleviate this challenge, as theoretically material would only need to be replaced in the cryopreserved backup once every century or more. On the other hand, these comparisons or rationalizations of whole collections can allow genebanks to reduce unnecessary duplicates if efficiencies need to be made in a particular collection. Additionally, a genebank manager may want to reduce the size of the collection if accessions are also found in another collection to make room for more diverse material.

**Figure 1:**
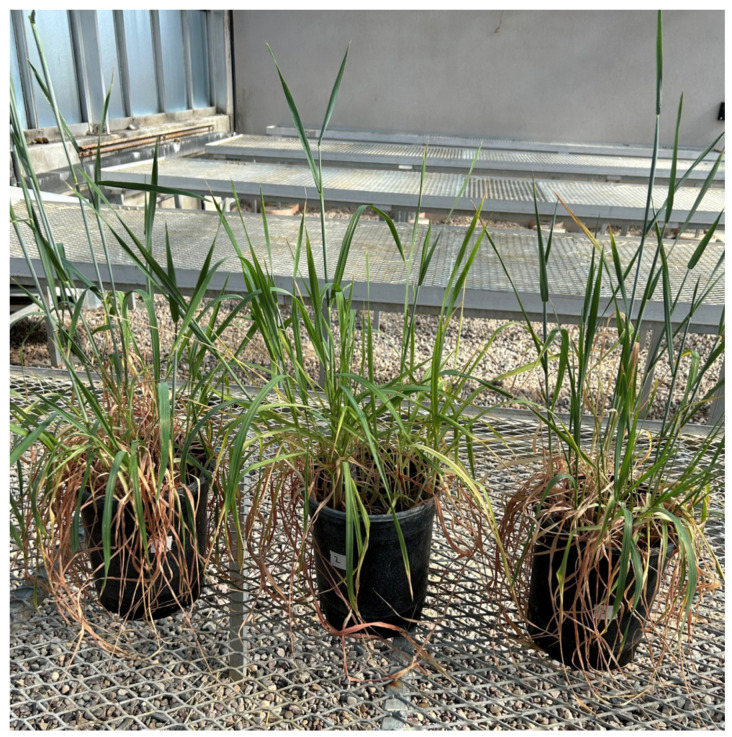
Different inventories of ‘Chinese Spring’ grown in the greenhouse to evaluate their morphology and collect leaf tissue for genotyping and identity verification.

Another strategy for monitoring genetic integrity issues in collections without extensive genotyping data and QMS genotyping strategies in place would be to perform targeted fingerprinting of the material in question in the genebank and compare it to another source of the same accession to determine if it matches with molecular markers followed by phenotyping. For example, recently at the National Small Grains Collection (USDA), a requestor received some seeds of ‘Chinese Spring” wheat. When the requestor grew out the material received, there were doubts if it was correct, and the grains genebank was contacted about the putative genetic identity issue. The National Small Grains Collection staff decided to investigate it further by planting out ‘Chinese Spring’ in the greenhouse in order to evaluate plant architecture, morphological characterization, and descriptor data and ensure that, during its growing cycle, it appeared to be typical of a Spring wheat type (Figure 1). In parallel, seeds of several regenerated inventories of ‘Chinese Spring’ wheat in the germplasm collection and the leaf tissue from the plants in the greenhouse growing for morphological characterization were collected and sent to a USDA lab for genotyping. Simple Sequence Repeat markers (SSRs) were chosen and run using the protocol from Schulthess et al., [24] on multiple seeds and leaf tissue of ‘Chinese Spring’ wheat. In addition, the lab included their source of ‘Chinese Spring’ to compare to the seeds and leaf material sent from the USDA National Small Grains Collection to help determine genetic identity. This analysis showed that the majority of the samples had matching alleles between the various sources of ‘Chinese Spring’ wheat and the plants all displayed similar morphological characteristics, helping to put to rest any concerns of mixed identity.

## 3. Use Cases

There are several reports in the literature of genotyping entire collections or a very large portion of a germplasm collection. Most of these studies were from seed-based, major crops housed in international genebanks. However, the genotyping of clonal collections has also occurred [6,7,22] and is discussed above. The return of investment (ROI) of clonal collections is potentially higher than that of seed collections due to the removal of genetic redundancy helping to reduce the overall operating costs of a clonal genebank. As time progresses, these large-scale whole-collection genotyping projects will become more common and likely lead to a significant amount of digital genetic data for accessions maintained in genebanks on top of the phenotypic and passport data that are already available. Molecular passports add value to genebank management by providing knowledge that goes beyond descriptors and passport data [25]. Genomic and genotyping data can play a role in increasing genebank management efficiency by allowing curators to make decisions about reducing redundancy in the collections [26] and understanding diversity or genetic gaps in their collections. It also helps define for seed collections the ideal number of plants that must be used for regeneration to lessen genetic drift and also avoid using an excessive number of plants to control the cost of regeneration [27]. In this section, an overview of some of the major efforts to genotype large crop collections is given.

### 3.1. Barley

**Figure 2:**
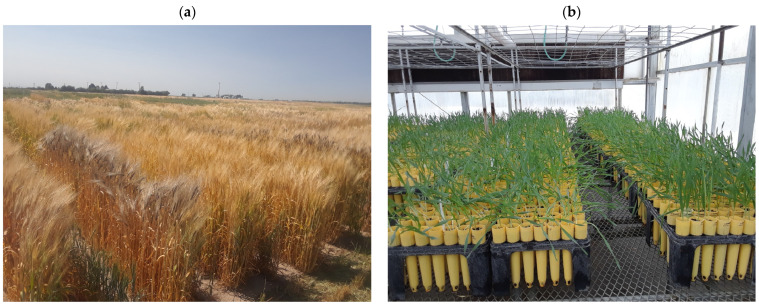

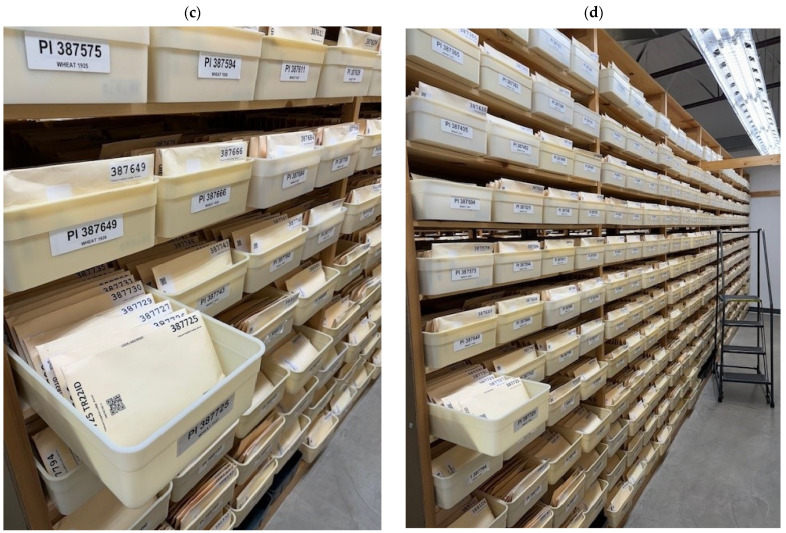
(**a**) A small grains field in Aberdeen, Idaho with material being regenerated and evaluated for the germplasm collection. The NSGC is one of the largest small grains collections in the world and currently maintains over 150,000 accessions of wheat, barley, oat, rice, rye, triticale, and related wild relatives. About 10,000 accessions need to be planted and processed each year to increase seeds for germplasm distributions and ensure collection viability. (**b**) Genotyping wild barley (*Hordeum spontaneum*). The seeds of each wild barley accession were grown in a cone and the young leaves from a single seedling were used for DNA extraction and genotyping. (**c**,**d**) Cold room of the genebank at the USDA Small Grains and Potato Germplasm Unit.

Barley (*Hordeum vulgare*, 2n  =  2x  =  14) is an important crop worldwide and was one of the ancient crops domesticated by humans. It was estimated that more than 466,351 barley accessions have been collected by over 47 major seed [28,29] including the USDA National Small Grains Collection (NSGC), which, as of writing, maintains 33,714 domesticated barley accessions and 2874 wild accessions obtained from over 100 countries (Dr. Harold Bockelman, Personal communication), [Figure 2]. Genotyping the barley collections can provide helpful information for both barley improvement and germplasm management. Muñoz-Amatriaín et al., [30] genotyped 2417 NSGC barley core accessions using 7842 SNP markers. Based on all pair-wise SNP comparisons, they identified 178 sets containing two or more genetically identical accessions for each and grouped the core accessions into five major subpopulations. The genotype data were further used to conduct genome-wide association studies (GWAS) and highly significant SNPs related to the heading date and other agronomic traits were detected. To gain more insights into the global population structure of barley and identify duplicated collections, Milner et al., [31] used genotyping-by-sequencing (GBS) to analyze 22,626 samples of cultivated and wild barley, including 21,405 accessions from the German Federal ex situ genebank at Leibniz Institute of Plant Genetics and Crop Plant Research (IPK) and 981 accessions from China and the Swiss national genebank of Agroscope. A total of 171,263 SNPs were detected and used to establish the molecular passport data for the collected germplasm. It was unexpected that 33% of IPK’s barley collection is likely duplicated. This large-scale study elucidated the global population structure of domesticated barley along with genetic redundancies and coverage gaps in their collection [31]. The high-density SNPs were used for GWAS analysis and the significant loci associations with barley yellow mosaic virus (BaYMV) and bymoviruses barley mild mosaic virus (BaMMV) resistance were detected. The genotyping data from 22,626 barley samples were next used to develop BRIDGE, a data warehouse and analysis tool for the genebank genomics of barley [32]. Through the BRIDGE website (https://bridge.ipk-gatersleben.de/, accessed on 1 January 2025), users can navigate between phenotypic traits and genetic and genomic information from GBS derived SNP profiles and correlate them with passport information in easy-to-understand graphical visualizations of the data. Genebanks serve key roles in preserving material into perpetuity that researchers can access to find needed traits. However, the genetic diversity of wild barley in germplasm collections is poorly understood. Fu and Horbach, [33] analyzed 269 core accessions of the wild barley (*Hordeum spontaneum*) collection using simple sequence repeat (SSR) markers and grouped these wild accessions into five clusters that revealed that accessions from Israel and Jordan were more genetically diverse than those of other regions.

### 3.2. Wheat

Wheat (*Triticum aestivum*, 2n = 6x = 42) is the most widely planted and traded food crop in the world. Since the 1990s, molecular markers have been used in wheat germplasm management in order to understand the genetic diversity and integrity of genebank accessions [34,35]. However, the earlier generations of molecular markers, including restriction fragment length polymorphism (RFLP), random amplified polymorphic DNA (RAPD), and simple sequence repeat (SSR) markers, were time-consuming and low-throughput. With the availability of the wheat genome sequences, high-throughput SNP genotyping tools became more popular in recent years. Bulli et al., [36] evaluated stripe rust resistance in 1175 NSGC wheat accessions and genotyped these with the wheat 9K-SNP iSelect assay. GWAS identified 127 resistance loci including five potentially new loci. Pfrieme et al., [37] screened 500 wheat accessions for wheat dwarf virus (WDV) resistance and genotyped 250 accessions with the 15 K iSelect SNP Chip. The genotyping data were used to conduct GWAS and identify QTLs associated with WDV resistance.

These studies indicated the importance of genotyping seed collections for exploiting new sources of wheat disease resistance and making this information available to the research community and breeders working on incorporating disease resistance into new releases. Genotyping by sequencing (GBS) was employed for over 7500 samples of wheat accessions in the IPK genebank along with 325 modern cultivars paired with whole genome sequencing (WGS) on 446 genebank accessions and scoring yellow rust resistance in the material [25]. The IPK wheat collection was also used to evaluate diversity, redundancy, mislabeling of accessions, genetic gaps in European genebank collections, selective sweeps, and alien introgressions introduced by breeding [38]. Seed banks also contain many unique traits such as anther extrusion (AE) which can improve cross-pollination and increase hybrid wheat seed production, important traits for breeding new varieties. Muqaddasi et al., [39] genotyped two doubled haploid wheat populations by crossing the accessions from the IPK genebank with a 15 K SNP array and identified a large QTL effect related to AE.

The identification of duplicated collections is critical for the efficient management of germplasm. Eliminating duplicates allows the genebank staff to focus on the characterization, regeneration, and distribution of novel material and conserves resources. Singh et al., [40] genotyped 1143 accessions of the wheat wild relative *Aegilops tauschii* using the GBS method and identified over 50% duplicated accessions both within and across genebank collections, which is a significant amount of duplication. To investigate the global genetic diversity of wheat germplasm and characterize useful genes for wheat improvement, Sansaloni et al., [41] genotyped approximately 79,191 accessions of domesticated wheat accessions and wild relatives originating from 109 countries using DArTseqTM technology, which is a high-throughput genotyping method and can detect allelic variations for genome-wide coverage. Over 300,000 high-quality SNPs and SilicoDArT markers were identified and mapped onto the reference genomes. The 56,342 hexaploid accessions and 18,946 tetraploid accessions were grouped into seven and four groups, respectively, and the 3903 accessions of wheat wild relatives were divided into 30 clusters. Their results also indicated that 2.4% of hexaploid and 4.4% of tetraploid samples are misclassified and demonstrated the value of genotyping data for identifying misclassification in germplasm collections. It is often difficult to visually find misclassifications of accessions especially when the phenotypic variation is subtle.

### 3.3. Soybean

The utilization of valuable germplasm has played a pivotal role in the genetic improvement of modern soybean (*Glycine max*). For example, the Chinese landrace (PI 88788) was widely used to control soybean cyst nematode which caused about a USD 32 billion yield loss between 1996 and 2016 in the United States [42]. Thus far, several studies have been conducted for the molecular characterization of soybean germplasm collections with different genotyping platforms. Thompson et al., [43] first analyzed the genetic relationship between 35 soybean genotypes using 281 RAPD markers. Song et al., [44] genotyped 19,648 accessions of wild and cultivated soybean preserved at the USDA Soybean Germplasm Collection with the SoySNP50K BeadChip. Based on the SNP comparisons, 8% of wild species (*Glycine soja*) and 9% of cultivated soybean were 100% identical to at least one other accession, which is a significant amount of duplication. However, if the cutoff for the redundancy of germplasm was 99.9%, 23% of cultivated soybean and 30% of wild soybean were likely duplicated.

With the availability of the soybean reference genome, whole-genome resequencing becomes a powerful method for understanding the genetic diversity of plant germplasm and identifying the genomic loci controlling important agronomic traits. Zhou et al., [45] resequenced 302 Chinese accessions of wild and cultivated soybean and conducted GWAS, and 13 previously uncharacterized loci associated with oil content, plant height, and other agronomic traits were detected. Recently, Fu et al., [46] genotyped 571 soybean samples, mostly from the Plant Genene Resources of Canada, using GBS and found that the samples originating from Canada can be clustered into three distinct groups. Forty samples were genetically duplicated in the collection. Mendonça et al., [47] genotyped 343 soybean lines from Brazil, North America, and Asia using GBS and revealed that the Brazilian soybean lines showed significantly lower genetic diversity than Asian accessions. Andrijanić et al., [48] genotyped European Germplasm with the SoySNP50K array and divided the 207 genotypes into eight subgroups that corresponded to the geographic origins of the cultivars.

### 3.4. Peanut

Peanut or groundnut (*Arachis hypogaea*, 2n = 4x = 40) is a legume used for oil and food globally. It is also an important cash crop for smallholder farmers in some developing countries. Otyama et al., [49] genotyped 787 accessions of the U.S. Peanut Core Collection using the Arachis_Axiom2 SNP array. Thirty-five accessions were identified as genotypically mixed, which was consistent with previous phenotyping results. Two hundred and fifty-three accessions were genotyped at least two times, and these results indicated that 86% of replicated pairings can be detected based on the genotyping data. Using a similar platform, Conde et al., [50] genotyped 1049 peanut breeding lines, varieties, and landraces collected from nine African countries. Over 70% of the collection can be unambiguously assigned into three peanut market types, and these genotypes were classified into *fastigiata* and *hypogaea* subspecies. By combining breeding traits and the genotyping results, a core Africa peanut collection composed of 300 accessions was developed, which provided valuable resources for peanut breeding in Africa.

Like many domesticated crops, cultivated peanut materials show very low genetic diversity; thus, it is helpful to transfer desirable traits from wild peanut species (most diploid) into the tetraploid cultivated peanut. Further, tremendous progress on introgression from wild peanuts has been made since the 1960s, and the peanut germplasm originally developed in the USA containing disease and pest resistance genes from the wild peanut species *A. cardenasii* GKP 10017 has been widely distributed in the world. However, the genetic contributions of the wild species were not well recorded and understood. Bertioli et al., [51] genotyped 383 cultivated peanuts from the US Core collection and 256 wild peanut accessions representing almost all of the described *Arachis* diploid species. By combining whole genome sequences and genotyping data with the Axiom Arachis Genotyping Array, Bertioli et al., [51] found that 82 registered peanut lines and cultivars contain chromosome segments of *A. cardenasii* GKP 10017. Further pedigree investigations revealed that 251 peanut varieties distributed in 30 countries have the genomic sequences from *A. cardenasii* GKP 10017. The work not only demonstrated the benefit and success of genotyping genebank material for tracking plant germplasm pedigrees but also highlighted the importance and necessity of international germplasm exchanges for ensuring global food safety.

## 4. Other Uses Beyond the Genebank—Driving Use of Collections Through Providing Data to Scientists

Genotyping seed collections provide precious resources for germplasm management, plant improvement, and basic research.

### 4.1. Germplasm Management and Conservation

An estimated 7.4 million accessions of germplasm are stored and have been collected or donated to 1750 global genebanks [2]. It is expected that more germplasm will be deposited in genebanks and the accession numbers will steadily increase. As each genebank has limited capacity in terms of its budget, staffing, and space for conservation, it is time-consuming and labor-intensive to store, process, and distribute large amounts of germplasm. Additionally, seed propagation and frequent handling and processing may cause contamination and affect the genetic integrity of collections. It is challenging and impossible to preserve all living plant genotypes in a genebank. Therefore, the removal of genetically redundant accessions both within and across genebanks is essential for genebank curators. However, the duplicated accessions cannot be correctly determined based on phenotype alone [30], nor based on just their passport data or molecular data. Two questions need to be answered for germplasm collections: (1) How many accessions are enough for maintaining the genetic diversity of each species?; and (2) What genotypes really needed to be stored? The genotyping data of germplasm collections allow scientists to establish molecular passport data for each accession, identify duplicated accessions, and understand the genetic diversity and population structures of current accessions. This information helps to collect and preserve rare or underrepresented genotypes or ecotypes and also assists genebank users in obtaining unique accessions suited for their needs.

### 4.2. Establishment of Core Germplasm Collection

One precondition for the efficient exploitation of germplasm is to fully evaluate and identify desirable traits in collections. As numerous accessions of germplasm are being stored in genebanks, especially for many major crops worldwide, very limited information is available for breeders to use to decide what accessions they can use for crop improvement and which accessions will give them the combining ability needed for the development of improved varieties. Also, it is impossible to incorporate all preserved accessions into plant breeding programs. One practical option is to establish and evaluate a core collection for each crop, with a limited number of accessions representing the majority of genetic diversity of the entire plant species in a smaller subset that represents around 10% of the entire collection. Based on morphological traits, core collections for many crops have been established, but further genotyping of these core collections has identified duplicated or mixed accessions [30,49]. Genotyping data from genebanks provide useful resources in establishing core collections for many important crops, including soybean [44,52,53] and peanut [50]. 

### 4.3. Molecular Mapping and Identification of Useful Genes with GWAS

Various high-density genotyping methods including SNP microarrays, GBS, and whole genome resequencing have been widely used to genotype genebank collections in order to provide invaluable resources for scientists by providing direct access to the genotyping data for identifying genomic regions significantly associated with numerous agronomical, physiological, and ecological traits, such as yield, grain quality, and disease resistance. Romay et al., [54] genotyped 2815 maize inbred accessions mostly preserved in the USDA genebank with GBS and generated 681,257 SNP markers across the whole genome. GWAS identified SNPs closely located to the known candidate genes controlling kernel color, sweet corn, and flowering time. The genotyping data were further used to conduct GWAS for Fusarium ear rot resistance, and seven SNP variants, each associated with small effects (1% and 3% of trait variation), were detected [55]. It should be noted that the efficiency of using genotyping data and GWAS for identifying candidate genes is impacted by many factors, including the number of molecular markers, the accuracy of phenotyping the collection, and the genetic architecture of targeted traits. As many plant genomes from many important crops have been sequenced and well annotated, the reference genomes can be used to develop additional markers and narrow down the genomic regions of candidate genes.

### 4.4. Marker-Assisted Introgression from Wild Species and Landraces

Plant breeders usually use very limited numbers of parenteral materials for their breeding programs, and numerous varieties or landraces with a lower yield but good adaptation to local environments are often excluded. Domestication and modern breeding have improved the crop yield and other important traits but also significantly decreased the genetic base of diversity in commercial cultivars. The narrowing of diversity in improved crops (genetic erosion) makes elite cultivars vulnerable to changing biotic and abiotic stresses. To develop new varieties with a high yield and better climate resilience, plant breeders will need to introduce useful genes, which have been lost in improved cultivars but are present in landrace varieties and/or wild relatives. However, many of these genes are difficult to uncover using conventional methods as they are hidden in poorly performing landraces or wild ancestors [56]. Further, there are often significant hybridization barriers to moving these traits from wild relatives into the cultivated form. Genotyping genebanks can illuminate the genetic diversity and population structures of collected germplasm but also identify molecular markers that are unique to specific groups of plant populations, landraces, and wild relatives and can assist with the introgression of new and desirable traits from underrepresented genotypes into elite germplasm [51].

## 5. Conclusions/Future Thinking

The utilization and exchange of germplasm is extremely important for sustainable crop improvement. Despite the large number of accessions of major crops that have been genotyped, it is challenging to compare the genotyping datasets and to evaluate the genetic diversity of all germplasm collected from different genebanks, as distinct genotyping methods were applied (different marker systems). To fully exploit the germplasm maintained in genebanks, it is critical to establish a global network for genotyping whole collections of each major crop using the same marker platforms or methods for the generation, storage, and analysis of genotyping data and enabling the datasets to be publicly accessed and fully used for comparisons within and between genebanks. The routine maintenance and updating of the genotyping databases are also needed. High-throughput genotyping platforms have been developed for many plant species. However, the costs of large-scale genotyping may still be unaffordable for many institutes in low- and middle-income countries. Thus, novel cost-effective, time-efficient, and high-throughput methods for DNA extraction and genotyping, along with effective collaborations, are highly encouraged for the exploration, evaluation, and utilization of the over seven million accessions stored in genebanks worldwide. Although crop domestication and modern breeding significantly improved crop yield and other important traits, these processes also reduced the genetic diversity of cultivated crops and made domesticated crop cultivars more vulnerable to evolving pathogens and a changing climate. It is necessary to use novel alleles or haplotypes identified in plant collections and expand the genetic variations of breeding materials for overcoming the breeding genetic bottleneck and developing crops with not only a high yield but also good quality and climate resilience.
